# The feasibility of using citizens to segment anatomy from medical images: Accuracy and motivation

**DOI:** 10.1371/journal.pone.0222523

**Published:** 2019-10-10

**Authors:** Judith R. Meakin, Ryan M. Ames, J. Charles G. Jeynes, Jo Welsman, Michael Gundry, Karen Knapp, Richard Everson

**Affiliations:** 1 Biomedical Physics Group, College of Engineering, Mathematics and Physical Sciences, University of Exeter, Exeter, United Kingdom; 2 Biosciences, College of Life and Environmental Sciences, University of Exeter, Exeter, United Kingdom; 3 Centre for Biomedical Modelling and Analysis, University of Exeter, Exeter, United Kingdom; 4 Medical Imaging, University of Exeter Medical School, University of Exeter, Exeter, United Kingdom; 5 Computer Science, College of Engineering, Mathematics and Physical Sciences, University of Exeter, Exeter, United Kingdom; University of Groningen, University Medical Center Groningen, NETHERLANDS

## Abstract

The development of automatic methods for segmenting anatomy from medical images is an important goal for many medical and healthcare research areas. Datasets that can be used to train and test computer algorithms, however, are often small due to the difficulties in obtaining experts to segment enough examples. Citizen science provides a potential solution to this problem but the feasibility of using the public to identify and segment anatomy in a medical image has not been investigated. Our study therefore aimed to explore the feasibility, in terms of performance and motivation, of using citizens for such purposes. Public involvement was woven into the study design and evaluation. Twenty-nine citizens were recruited and, after brief training, asked to segment the spine from a dataset of 150 magnetic resonance images. Participants segmented as many images as they could within three one-hour sessions. Their accuracy was evaluated by comparing them, as individuals and as a combined consensus, to the segmentations of three experts. Questionnaires and a focus group were used to determine the citizens’ motivation for taking part and their experience of the study. Citizen segmentation accuracy, in terms of agreement with the expert consensus segmentation, varied considerably between individual citizens. The citizen consensus, however, was close to the expert consensus, indicating that when pooled, citizens may be able to replace or supplement experts for generating large image datasets. Personal interest and a desire to help were the two most common reasons for taking part in the study.

## Introduction

Automatic segmentation methods are important in many medical and healthcare research areas. They are often an essential component of computer aided diagnosis software which can be used clinically to increase efficiency, help less experienced clinicians, and improve diagnostic accuracy [1]. They are also important for realising recent technological advances in, for example, radiotherapy [2] and the use of rapid prototyping in surgery [3].

Many methods for automatic medical image segmentation have been developed but few have progressed much past the initial development stages. One reason for this is the low availability of data that can be used to train and test the methods [4]. Obtaining sufficient example data for training and testing is difficult because human experts are required to generate these data; experts are often expensive and may not have the time to perform the amount of analysis required. Furthermore, as there is often a subjective element to the analysis, the use of a single expert may introduce bias, exacerbating the difficulty of obtaining data to robustly train and test a computer.

Citizen science may provide a solution to the problem. The engagement of the public to help inform, design and undertake research tasks has increased greatly over the last two decades, particularly in astronomy and bioscience, due to emergence of online platforms [5]. Although the use of citizen science has lagged considerably in the medical fields [6], we hypothesise that citizens would be motivated to engage with medical image segmentation as it has a potential future benefit for their own healthcare. We also hypothesise that, since studies have shown that novices can be trained to identify disease in medical images with a diagnostic sensitivity and specificity that is close to expert performance [7], it may be possible that with some appropriate training, citizens could replace or supplement experts when generating data for the development of automatic methods.

The primary aim of our project was therefore to explore the feasibility of using citizens to provide segmentation data that could be used to train and test computer algorithms. This aim was achieved by recruiting citizens to segment spinal anatomy from magnetic resonance images and comparing their segmentations to those from three experts. The secondary aims were to determine the motivation of citizens taking part in the study and to produce recommendations for the use of citizens in similar future studies. This was achieved by using public involvement to help design and evaluate the study and by using a combination of questionnaires and a focus group to assess the motivation and future study requirements.

## Methods

### Public involvement

Public involvement (PI) was used to explore the concept of the study and design the study protocol. PI was planned and budgeted for at the grant writing stage to enable participants’ travel expenses and a thank you payment of £25 to be offered. Two members of the public who belong to the Centre for Biomedical Modelling and Analysis public advisory group (MAGPIES http://www.exeter.ac.uk/cbma/getinvolved/magpies) were integral to this early planning and worked closely with the researchers to develop the PI plans.

Members of the PI group were recruited from existing patient and public networks and personal contacts via a flyer inviting people to attend the first PI workshop. At the first half day workshop participants met the research team and were introduced to the studies’ aims and objectives. The researchers were keen to explore whether the “Citizen Science” concept of the study and its specific activities (segmenting medical images) would be appealing to the public and if so, who might be the types of people willing and interested to get involved. The discussion then moved to more specifics around the content and protocols for the study. The group discussed the types of public who might be approached as participants, the level of expertise needed, and how these might be reached. The group were involved in ensuring the participant information sheets and consent forms were written in plain English and were key in rephrasing the inclusion and exclusion criteria for potential participants.

In the second workshop the key results of the study were fed back to the group including the results of the focus group held with people who had been involved as project participants. The nature of future studies and the important aspects to incorporate into these were discussed.

### Image dataset and segmentation software

The images used in this study were acquired for a previous study [8]. They were MR images of the lumbar spine acquired from 15 volunteers who had given their informed consent for their image data to be used in future studies. Scans comprised 60 axial slices (one scan contained only 20 slices) with an in-slice resolution of 0.49 mm, slice thickness of 4 mm, and slice spacing of 6 mm. The scans had been acquired using a T1-weighted sequence and a spine receiver coil (repetition time = 414 to 497 ms, echo time = 8 ms, flip angle = 90^o^, number of signal averages = 4). A random selection of 10 slices were selected from each scan (excluding slices that were outside the lumbar region) leading to a dataset of 150 images.

**Fig 1:**
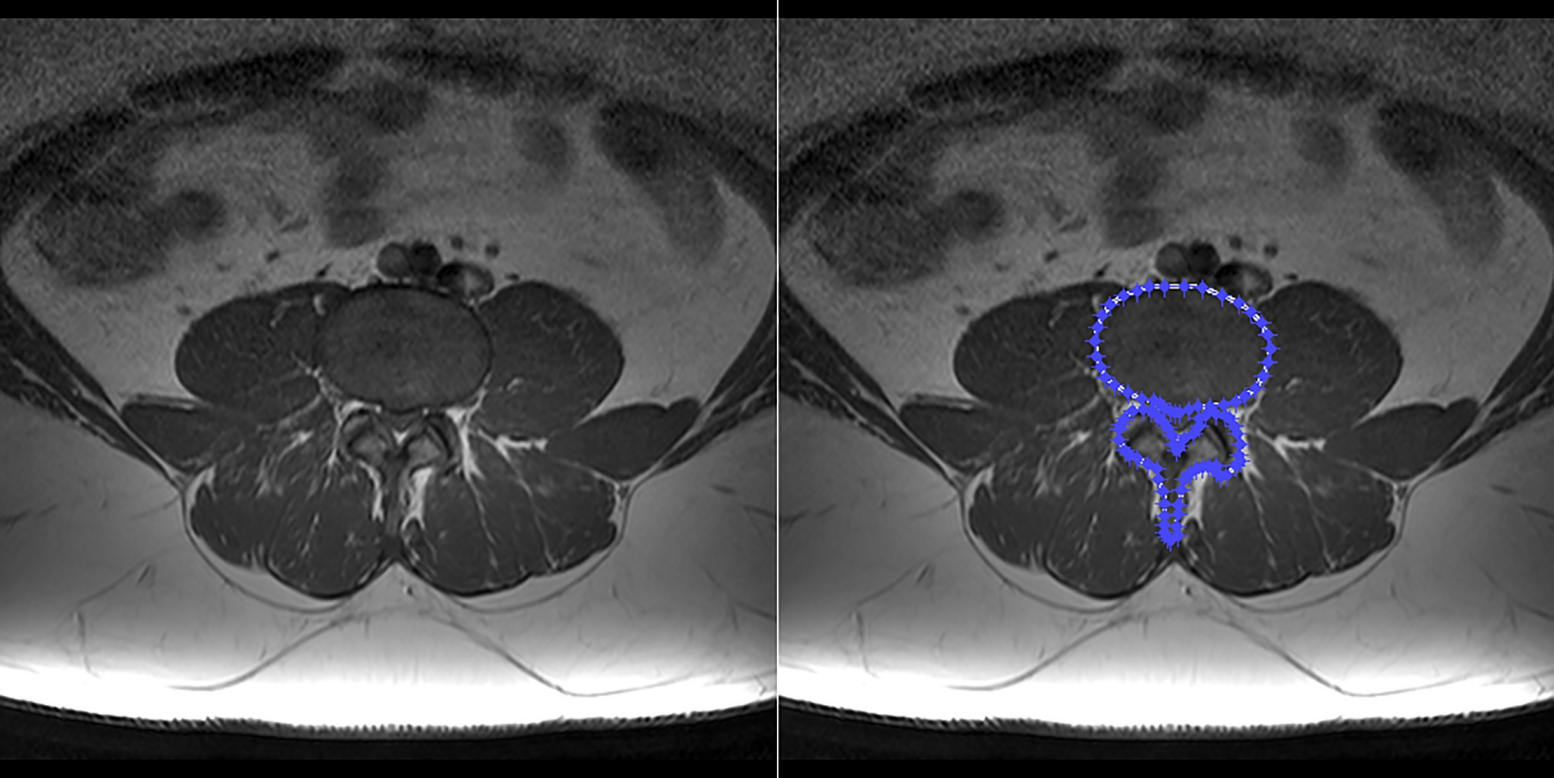
MR image and segmentation. An MR image is shown on the left and the same image is on the right with the vertebral bone outlined.

Software tools for drawing around the vertebral bone on the images were written in Matlab; the code is found at www.github.com/charliejeynes/citizen_segmentation. These tools presented the user with a randomly selected, and previously unanalysed, image from the dataset. The randomisation ensured that users were unlikely to be presented images in the same order as another user and thus helped provide even coverage of segmentations across the image dataset even if not all users segmented all images. The contrast of the image and the magnification could be changed to suit the users’ preference. It then allowed the user to place points on the image to define the edges of the vertebrae; an example is shown in Fig 1. Multiple regions could be defined and amended after which the user could submit their analysis of that image. Users were not given the option to skip images.

### Citizen participants

Citizen participants were recruited using social media, local Patient and Public Involvement (PPI) groups, word of mouth, and adverts at the University of Exeter campuses. Inclusion criteria were adults (over 18 years) in the local area that had a steady hand and normal eyesight (with or without glasses). Exclusion criteria were current or previous professional involvement in medical imaging (e.g. radiologist or radiographer). All participants gave written informed consent for their participation and were offered a payment of £25 and reimbursement for travel expenses. Ethical approval for the study was granted by the ethics committee of the College of Engineering, Mathematics and Physical Sciences, University of Exeter.

### Experts

Experts comprised three individuals: two radiographers and a researcher with extensive experience of segmenting spinal anatomy from MR images. The experts segmented all 150 images using the same software tools as used by the citizens. They performed this on their own computers using image viewing conditions of their preference; preliminary assessment on five images found no systematic differences induced by using different computers and viewing conditions.

### Data collection

The citizen participants were asked to attend three sessions. In the first session a questionnaire was used to ascertain their age, sex, current and previous occupation, and their motivation for taking part in the study. Their free-form answers concerning motivation were analysed using thematic analysis.

They were then given information about the image data (including information on lumbar anatomy and the features that could be seen in an MR images) and training on how to use the software tools. They were shown example segmentations and kept these to refer to whilst they were completing the segmentation task; these example segmentations were of images not included in the 150 image dataset.

The segmentation task was performed in the same room each time and viewing conditions were kept the same during the study (there were no windows and lighting was controlled to a set-level on all study session). Participants were positioned so that they could not easily see what each other was doing. During each session, the participants performed as many segmentation as they were able over a one hour period; participants were allowed to rest freely and a mandatory refreshment break was given approximately half-way through the session.

For three participants, data were only available for two out of three sessions. For participant 17 this was because they completed 149 images in their first two sessions and so did not return for a third session; for participants 09 and 20 this was because the software crashed resulting in data loss. In addition to this, data from the third session for participant 02 included images that had already been analysed in a previous session. These duplicates were included when considering the number of images that the participant had segmented but were excluded when considering the accuracy of the segmentations.

On completing their third session, citizen participants were given a questionnaire that asked how easy or difficult they had found it to identify the vertebral anatomy and to use the software tools. Their response was captured on a four point scale (very easy–easy—quite difficult—very difficult). The questionnaire also asked participants for suggestions on how to improve the study. Their free-form comments were analysed using thematic analysis and categorised into themes.

### Data analysis

Coordinates from image segmentation for participants and experts were analysed using the Python programming language. Raw data were manipulated using the pandas package and DICOM images were processed using pydicom. Segments were calculated using a Python implementation of the scanline algorithm. Statistical analyses were performed by using the numpy and scipy packages. Visualisations were created with the matplotlib package.

For both citizen and expert segmentations, consensus segmentations were calculated. These were defined as the segment that contained regions segmented by >50% of citizens or experts. Additional analysis was performed to assess the effect of changing this threshold to 25% and 75%.

Accuracy between segmentations was quantified using the Dice similarity score. This is a coefficient describing the amount of overlap between two regions, A and B, relative to their combined size:

$$Dice\left(A,B\right)=\frac{2\left|A\cap B\right|}{\left|A\right|+\left|B\right|}$$ (1)

### Focus group

Participants who had completed the study were asked if they would take part in a focus group to explore their experiences of the study. Four participants took part in a one-hour session. During the focus group a semi-structured approach, with trigger questions and prompts, was utilised to guide the conversation within the areas required to explore their experiences, thoughts and opinions. The session was audio recorded and then transcribed, using an intelligent verbatim approach, by an experienced transcriber with the transcription being checked by the researchers who ran the focus group. Thematic analysis was undertaken to compress the transcript into themes which are presented in the results. Participants taking part in the focus group were offered an additional payment of £10 and reimbursement of travel expenses.

## Results

### Public involvement

Eight members of the public constituted a Public Involvement (PI) group and represented a broad range of ages, backgrounds and interests. They were supportive of the concept of using citizens to generate data for developing automatic segmentation methods and thought that the study would be attractive to a broad range of people. The group discussed the types of public who might be approached as participants, the level of expertise needed, and how the participants might be reached. The group helped design the study protocol including advising on the training that should be given to the participants.

The group were also involved in ensuring the information sheets and consent forms given to participants were written in plain English and made suggestions on how to phrase the inclusion and exclusion criteria for potential participants. A particular outcome of these discussions included the use of medical imaging terminology, particularly the word “segmentation” which was felt to be too obscure. As a result of this discussion, the phrase “tracing around” was used in the participation information sheets and consent forms both in the title and in explanation of the project.

### Participant demographics and motivation

A total of 38 people responded to recruitment adverts and expressed interest in participating in the study. Nine of these were unable to participate due to either being ineligible, unable to attend scheduled study sessions, or not responding after expressing their initial interest. The remaining twenty-nine were recruited as participants and completed the study. The median age of the participants was 40 years (range 18–73 years). The majority of participants were female (22 females, 7 males). Twelve of the participants were students (undergraduate or postgraduate), 11 were in paid employment, and 6 were retired or in non-paid employment.

The motivation that the 29 participants gave, on the pre-study questionnaire, for taking part in the study fell into three main themes: personal interest (n = 25, 86%), a desire to help (n = 9, 31%), and the financial incentive offered (n = 5, 17%). These findings were echoed by the four participants who took part in the focus group to explore their experience of the study in more depth.

… *it is really interesting because when do we ever get the opportunity to see these images so there is a natural curiosity…*

… *I rather feel that we’re doing something like that*, *we’re really helping in some way or other…*

### Segmentation performance

**Fig 2:**
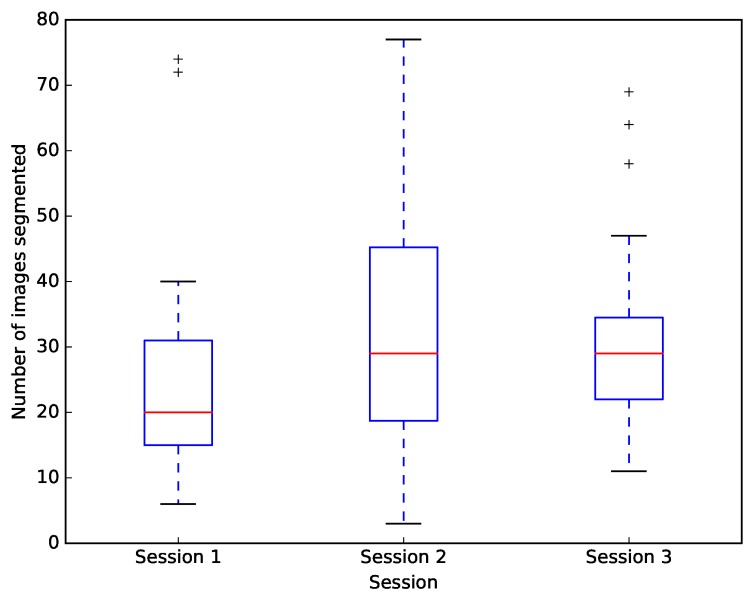
Number of images segmented by citizens at each session. Data is for all 29 citizen participants. Box plots show the median (red line), the lower to upper quartiles (blue boxes), range (dotted blue lines) and outliers (+ symbols).

Each citizen participant segmented 85 ± 43 (mean ± standard deviation) of the 150 images in the image dataset. The median number of images segmented per session increased from 20 in the first session to 29 in the second and third sessions (Fig 2). The increase from session one to session two was statistically significant (Mann Whitney U = 286, p = 0.028). The change from second to third was not significant (U = 375.5, p = 0.486).

These results suggest that participants became slightly quicker after the first session; either as their familiarity with using the software increased or as they began to recognise the features in the images they were segmenting. Comments from the focus group indicated that as they progressed, some of them started attributing their own classification system to the vertebrae by describing them as having the shape of things they were already familiar with (e.g. ‘Darth Vadar helmet’ or a ‘Jellyfish’). However, they also felt they slowed down in the third session because they started thinking more about what they were doing.

*You do get your eye in*, *by week three I think I was pretty good at clicking*.

*I found week three harder than week two*. *I found I was thinking more about what I was doing*…

“*…and then I wasn’t sure why I was choosing that it was one or the other and that then made me slower because I was engaging more with it and wishing I had studied what I was looking at more…”*

**Fig 3:**
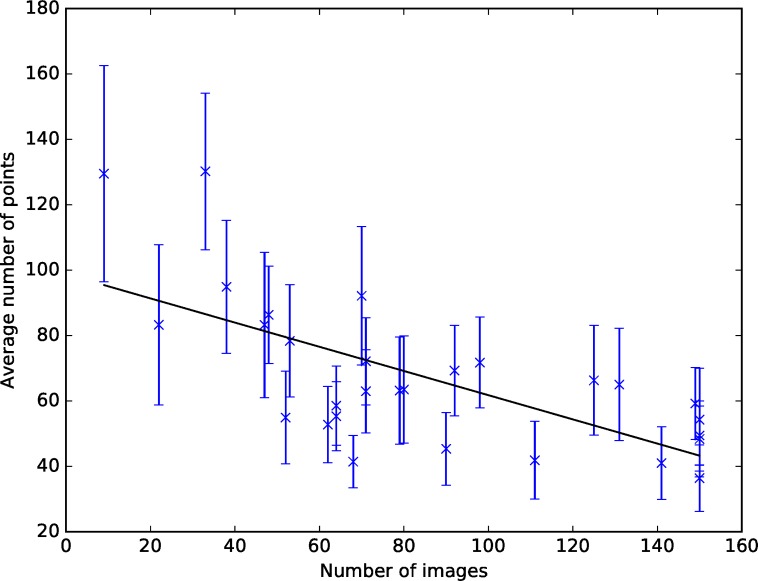
Number of points versus number of images analysed. Error bars show the standard deviation.

The mean number of points each participant used to define the outline of the vertebrae varied from 36 to 130. This had a significant negative correlation (Pearson’s r = -0.675, p < 0.001) with the number of images analysed (Fig 3), demonstrating that participants who placed many points analysed fewer images, possibly because placing more points made them slower.

Age had a small effect in that there was a tendency for the number of points per image to increase, and the number of images analysed to decrease with age but these correlations were not statistically significant.

### Segmentation accuracy and precision

**Fig 4:**
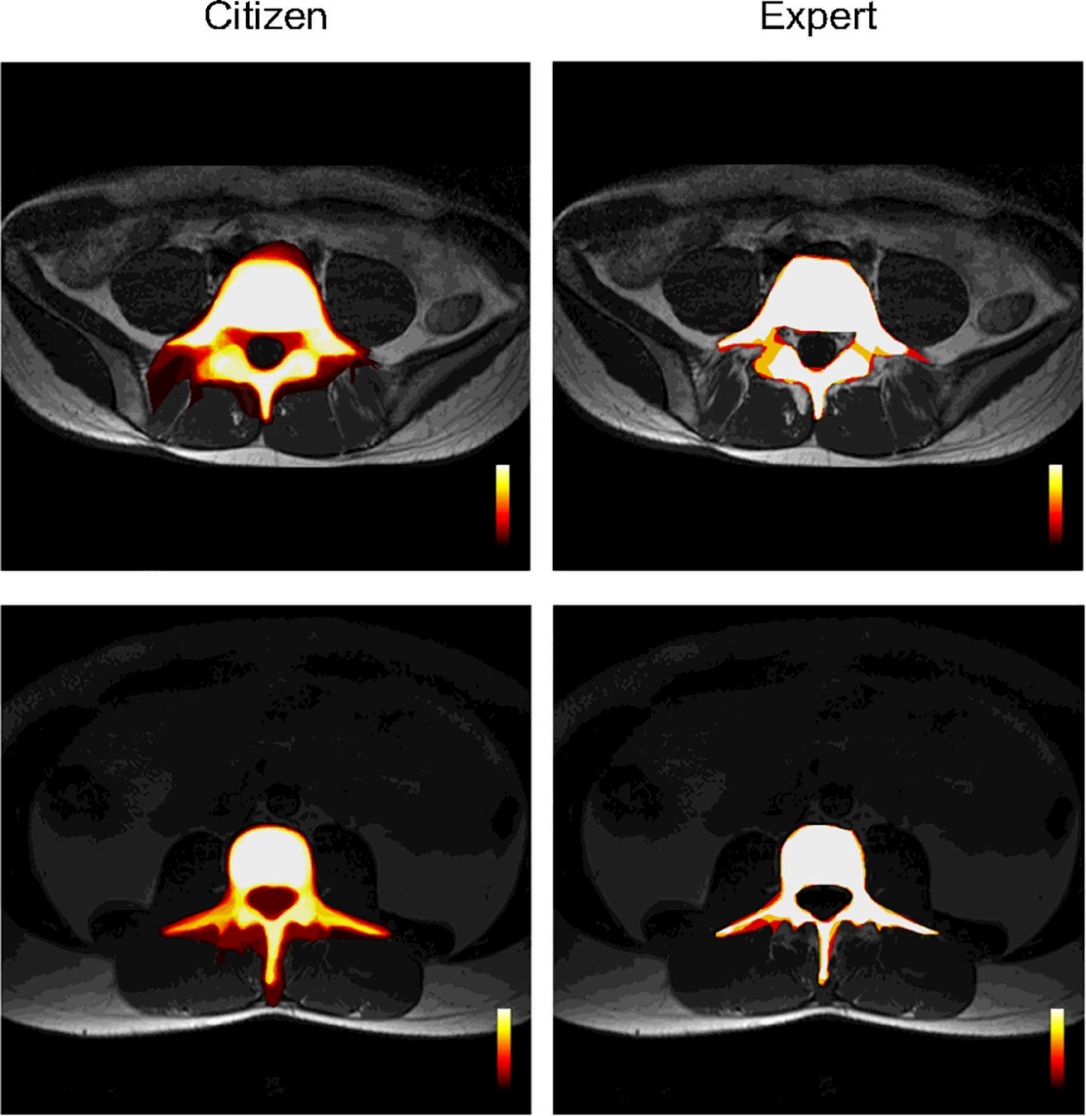
Consensus maps. Example consensus maps from citizens (left) and experts (right). The colour scale represents the number of segmentations and ranges from no segmentations (dark red) to all segmentations (white).

The segmentations from the citizens were combined to produce consensus maps (Fig 4) ranging from 0 (pixel not included in any segmentations) to 1 (pixel included in all segmentations). This was repeated for the segmentations from the three experts. The consensus segmentation was then defined (separately for citizens and experts) as the region where the consensus was greater than 0.5.

**Fig 5:**
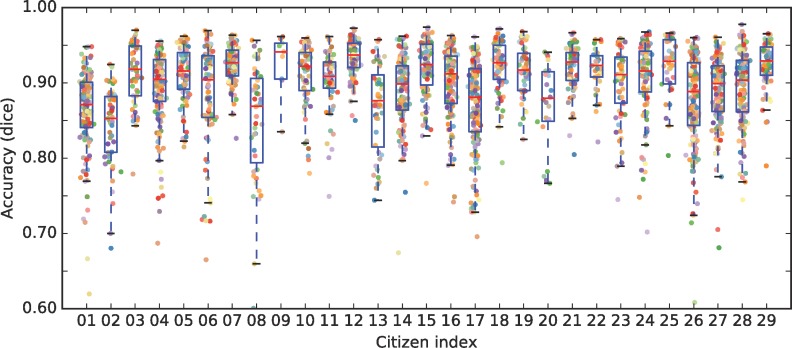
Segmentation accuracy by citizen. The plots show the accuracy (Dice coefficient) for each citizen, compared to the expert consensus (a score of 1 indicates exact overlap with the experts). Each dot represents a single image. Box plots show the median (red line), the lower to upper quartiles (blue boxes) and range (dotted blue lines).

The accuracy of the citizens was evaluated by comparing their individual segmentations to the expert consensus segmentation and quantifying the overlap using the Dice similarity score (a coefficient that describes the amount of overlap between two regions) which ranges from 0 (no overlap) to 1 (perfect overlap). This showed that the segmentation accuracy (considering expert consensus as the ground truth) varied between citizens and images but that most citizens achieved a Dice similarity score above 0.8 for the majority of images they segmented (Fig 5). Across the images, the median citizen segmentation accuracy (red line in (Fig 5)) ranged from 0.80 to 0.96 (mean 0.91, standard deviation 0.03). Age was negatively correlated with accuracy (Pearson’s r = -0.425, p = 0.02) but the effect was small with a reduction in Dice score of only 0.005 per decade.

**Fig 6:**
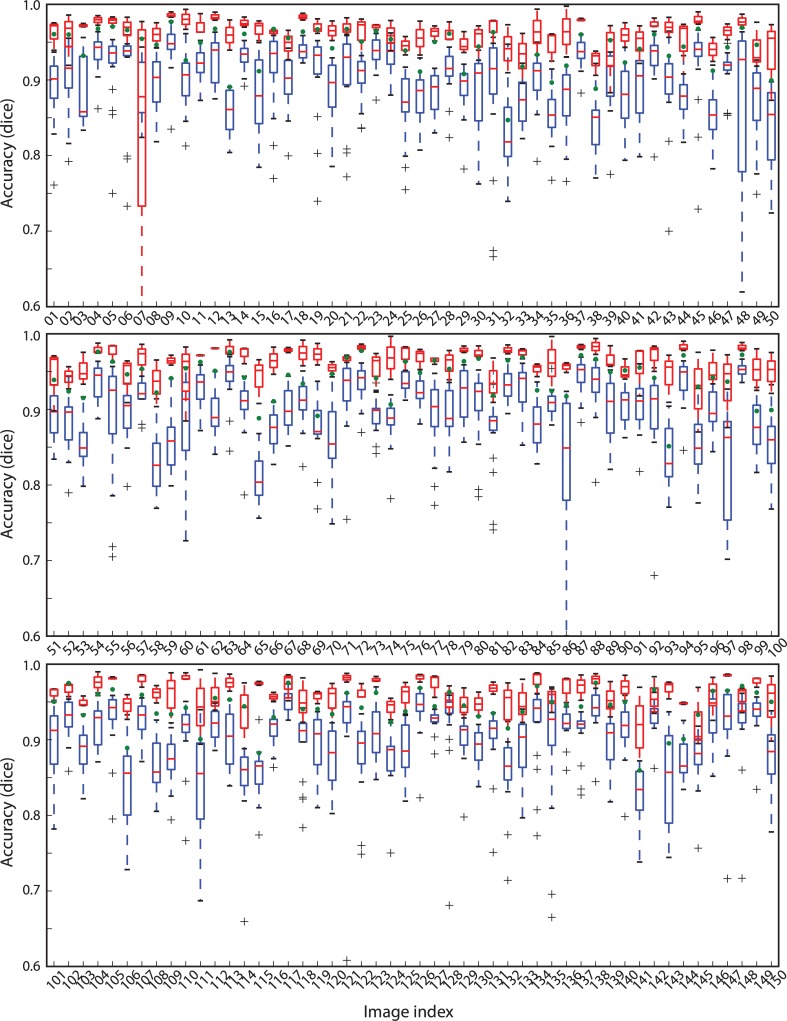
Segmentation accuracy by image. The plots show the accuracy (Dice coefficient) for every image in the data set. The blue box plots summarise the individual citizens compared to the expert consensus. The green dots show the citizen consensus compared to the expert consensus. The red box plots show the agreement of the experts (pairwise comparisons).

The accuracy of the citizen consensus was then compared to the expert consensus (Fig 6). Across the images, the citizen consensus accuracy (green dots in (Fig 6)) ranged from 0.84 to 0.98 (mean 0.94, standard deviation 0.03). This was generally higher than that of the individual segmentations (blue box plots in (Fig 6)). Furthermore, the citizen consensus segmentation accuracy was within the inter-expert agreement (calculated from pairwise comparisons) for many of the 150 images (red box plots in (Fig 6)). Across the images, the median expert segmentation agreement ranged from 0.90 to 0.99 (mean 0.96, standard deviation 0.01). Using a lower or higher threshold to define the expert and citizen consensus had little effect on the results with the mean citizen consensus accuracy of 0.94 (standard deviation 0.02) for a threshold of 0.25 and 0.93 (standard deviation 0.05) for a threshold of 0.75.

### Participant evaluation of the study

In their evaluation of the study, most participants found the software used to segment the images easy to use (Table 1) but had ideas on how it could be improved. In particular, several participants said that they did not like having to repeatedly click a mouse button to place points on the images and suggested that using a stylus or touchscreen would be preferable. Although some of the focus group participants concurred with this, it was also noted that a choice of methods may be preferable.

*I couldn’t have done it with a touch pad*…*I was fine so I think you’d have to give people the option*.

**Table 1:** Participant evaluation. Distribution of participants (n = 29) rating the difficulty of using the segmentation software tools and identifying the vertebra in the images.

	Using segmentation software	Identifying the vertebra
**Very easy**	17%	0%
**Easy**	52%	31%
**Quite difficult**	24%	62%
**Very difficult**	7%	7%

The identification of the vertebrae in the images was rated by most participants as being quite difficult (Table 1) and around half the participants (n = 15, 52%) suggested that additional training would have helped. Suggestions included having more examples to refer to and feedback on how well they were doing. This was further explored in the focus group and post-study PI where suggestions were made that training could include a test where participants had a go at segmenting an image and then received feedback on whether they had done it appropriately.

## Discussion

### Summary

Our study demonstrates the feasibility of using citizens to provide segmentation data that could be used to train and test computer algorithms for automatic segmentation of medical image data. A great deal of effort has been placed in recent years in developing automatic methods for image segmentation. The purpose of these methods can be to make a clinical decision from image data (e.g. [9] or as a step for planning treatment (e.g. [2]) or surgical aids (e.g. [3]). For these efforts to be translated into clinical or research benefit, the machine learning algorithms need to trained and tested on many example images to ensure that they have captured the variation in the population. Without adequate data, the development of these algorithms is limited but many studies are constrained to small datasets as it is time consuming and expensive for experts to perform the segmentations required to produce the training data. Using citizens to replace or supplement experts thus has the potential to allow much larger image datasets to be used for training and testing. This could have a tremendous impact in the field if our results are confirmed in a larger study.

### Segmentation performance

Segmentation is an inherently subjective task leading to differences between individuals as demonstrated in the variability in both the citizens and the experts. For most images in our study the variability in the segmentation data from the citizens was large. The experts were more consistent in their segmentation, which might be expected given that all three experts had knowledge and previous experience of medical imaging and spinal anatomy. This would allow them to recognise and deal with an unexpected features in the anatomy. On the other hand, the smaller variability may be due to the smaller number of experts (3 versus 29 citizens) since other studies have found little difference in precision between experts and either citizens, [10] or varying levels of expertise [11].

The mean expert agreement Dice score of 0.96 was similar to previous studies that have found a Dice scores of 0.9 for the aorta [12], 0.81 for white matter in the brain [13], and 0.80, 0.92 and 0.88 for breast, brain tissue and brain tumour respectively [14]. The better agreement in the anterior regions of the spine (the vertebral body) compared to the posterior regions (posterior elements) (Fig 4) is consistent with the findings of Deeley, Chen (11) that agreement tends to be higher when segmenting large round structures and lower when segmenting small thin structures.

The performance of the citizens as individuals (mean Dice score of 0.91) and as a consensus (mean Dice score of 0.94) was comparable to the expert agreement. This suggests that, despite the citizens reporting that they found it quite difficult to identify the vertebrae, they were able to perform the segmentation task reasonably well. In our study, the task involved placing points to define the edges of the vertebrae. All the images were from healthy people and so, although vertebral anatomy varies between people and at different levels along the spine, the task did not involve significant anatomical variation or pathology. Although citizens can be trained to recognise patterns relating to anatomy on an image, they are less likely than experts to consider any significant abnormalities or disease.

Accuracy of individual and consensus citizen segmentation was evaluated by comparison to the expert consensus segmentations. The consensus segmentations were produced by thresholding the consensus maps at a value of 0.5, which represents the majority vote. Using a threshold of 0.5 has been used to combine individual segmentations for training automatic methods [15] although higher threshold values have also been used and more sophisticated methods exist to eliminate outliers and weight results towards more precise individuals [15]. Although the choice of threshold is important for training automatic segmentations, it may be less so for comparing citizens to experts since the threshold affects them similarly. Analysis of our data with a lower (0.25) and higher (0.75) threshold changed the calculated accuracy of the citizens by only a small amount.

### Participant demographics and motivation

Our participant’s strongest motivations for taking part in the study were personal interest and a desire to contribute. Previous studies have also found these two reasons to be important to participants but, contrary to our study, tend to find that the desire to contribute is most common and personal interest is second [16–18]. This may be because other studies have had a clearer link between participation and a specific research outcome such as finding a cure for a medical condition. It could also be because many of our participants were recruited from the medical school campus of a university and participants who engage in scientific activities as part of their daily lives are more likely to report personal interest as their primary motivation for taking part in citizen science research [17].

Many of our participants, studying or working at a university, are likely to have a higher educational level than the general population. Data from other citizen science projects suggest that this is representative of the educational level of people who take part in citizen science projects [16, 18]. However, recruiting from a medical school campus means that our student participants in particular may have had greater than average knowledge of anatomy (although it is unlikely that they are familiar with how anatomy appears on an MR image).

The majority (76%) of the participants were female whereas citizen projects in astronomy have found the majority (80%) to be male [16]. In terms of age we attracted a range of ages similar to other studies [16]. Although age was found to have a negative effect on the number of images analysed and the accuracy of the segmentations, these effects were too small to be an important consideration for future studies.

### Recommendations for citizens in medical image segmentation

Our study participants were trained by providing an explanation of the MR image and spinal anatomy together with examples of expert segmentations which the participants were able to refer to whilst they performed their own segmentations. Participants said the examples were helpful but that more were required to cover the full range of anatomical variation in the image dataset. Several also suggested that some form of feedback, perhaps showing how their segmentations compared to an expert, would allow them to evaluate their performance and either give them reassurance or prompt them to adjust. A study on assessing bee species highlights the importance of feedback in citizen science and shows that adding information to promote learning improves accuracy [19]. This approach could be further extended using a combined citizen-computer approach where automatic methods suggest a segmentation for the citizens to modify, allowing the automatic methods to learn and improve in a iterative way.

The scale of involvement of citizens can vary but is described by Bonney, Ballard [20] as falling into three broad categories: contributory, collaborative and co-created. We used an approach which incorporated both collaboration and co-creation since the public involvement included helping to design the study, collecting and analysing data, and in discussing the results and future steps. The involvement was seen as essential from the beginning of the study and should be included in similar studies in the future. It proved key in ensuring the appropriate use of language that the public could understand provided useful feedback for subsequent development of our research.

## Conclusion

Our study has investigated the feasibility of using citizens to segment anatomy from medical images. Our results found citizens were motivated to take part in this type of activity and that their consensus segmentation was similar to that from experts. These results suggest that citizens could be used, with appropriate training, to replace or supplement experts to provide data for training and testing computer algorithms. The ability to do this economically across much larger image datasets than is usually possible would improve the development of automatic segmentation methods.
